# Association between genetic variants and development of antibodies to infliximab: A cross-sectional study in Chinese patients with Crohn’s disease

**DOI:** 10.3389/fphar.2023.1096816

**Published:** 2023-01-16

**Authors:** Kouzhu Zhu, Xiaoliang Ding, Zhiyao Chen, Qinhua Xi, Xueqin Pang, Weichang Chen, Liyan Miao

**Affiliations:** ^1^ Department of Pharmacy, The First Affiliated Hospital of Soochow University, Suzhou, China; ^2^ College of Pharmaceutical Sciences, Soochow University, Suzhou, China; ^3^ Institute for Interdisciplinary Drug Research and Translational Sciences, Soochow University, Suzhou, China; ^4^ Department of Gastroenterology, The First Affiliated Hospital of Soochow University, Suzhou, China; ^5^ National Clinical Research Center for Hematologic Diseases, The First Affiliated Hospital of Soochow University, Suzhou, China

**Keywords:** infliximab, anti-drug antibodies, genetic variants, drug-tolerant enzyme immunoassay, Crohn’s disease

## Abstract

**Aims:** Genetic variants increase the susceptibility to anti-drug antibodies (ADA) in response to anti-TNF therapy in chronic inflammatory diseases. However, little is known about genetic variants in Chinese populations. This study aimed to identify genetic variants contributing to the risk of the development of antibodies to infliximab (ATI) in Chinese patients with Crohn’s disease (CD).

**Methods:** CD patients (n = 104) treated with infliximab (IFX) during the maintenance therapy were enrolled in this cross-sectional study. ATI was assessed by an in-house developed drug-tolerant ELISA method. ATI titers of 1:20 and ≥1:60 were considered a low titer and a high titer, respectively. Thirteen types of single nucleotide polymorphisms (SNPs) within 13 genes involved in the immune process, the susceptibility to chronic inflammatory diseases, cytokines and apoptosis pathways were investigated.

**Results:** The median trough levels of infliximab (TLI) in patients with clinical remission (CR) were higher than those in patients without CR (3.80 vs. 1.50 μg/mL, *p* < .001). The median TLI in patients with high-titer ATI was significantly lower than that in ATI-negative patients (1.15 vs. 4.48 μg/mL, *p <* .001) or those with low-titer ATI (1.15 vs. 2.95 μg/mL, *p* = .03). The HLA-DQA1*05 rs2097432 GG and GA genotypes were more frequent in patients with ATI (GG and AG vs. AA, 27/38 = 71.05% vs. 29/66 = 43.94%, OR 2.94, 95% CI 1.19–7.30, *p* = .02). Patients carrying the CC and AC genotypes of rs396991 in *FCGR3A* were associated with a higher frequency of ATI formation (CC and AC vs. AA, 37/57 = 64.91% vs. 19/47 = 40.43%, OR 2.94, 95% CI 1.24–6.96, *p* = .01). According to the number of variants in rs2097432 and rs393991, patients with two variants had a higher proportion of producing ATI (two variants vs. no variant, 17/21 = 80.95% vs. 9/30 = 30.00%, OR 9.92, 95% CI 2.59–37.87, *p* = .001; single variant vs. no variant, 30/53 = 56.60% vs. 9/30 = 30.00%, OR 3.04, 95% CI 1.18–7.88, *p* = .02). No association was found between other SNPs and ATI production.

**Conclusion:** Rs2097432 in HLA-DQA1*05 and rs396991 in *FCGR3A* are associated with ATI production in Chinese patients with CD. A pharmacogenomic strategy could help with the clinical management of CD.

## Introduction

Crohn’s disease (CD) exhibits periods of remission and aggravated inflammations in the gastrointestinal tract. Infliximab (IFX), a chimeric monoclonal antibody against tumor necrosis factor alpha (TNF-α), has profoundly improved therapeutic outcomes (Torres et al., 2020). Many studies have demonstrated the correlations between trough levels of infliximab (TLI) and therapeutic targets (Ward et al., 2017; Yarur et al., 2017; Papamichael et al., 2018). Up to 30% of individuals with CD experience loss of response in the maintenance phase due to an inadequate TLI (Zhang et al., 2019; Greuter et al., 2020). A major contributor to subtherapeutic levels of IFX is immunogenicity, which refers to the development of anti-drug antibodies (ADA) (Hemperly and Vande Casteele, 2018).

A number of factors were identified for ADA development, including genetic predisposition, antibiotic use differentially and formation of drug-target complexes (Moss et al., 2013; Bar-Yoseph et al., 2019; Gorelik et al., 2022). Increasing evidence suggests that the variation in the *HLA-DQA1* gene involved in aberrant adaptive immune responses is relevant to the development of antibodies to infliximab (ATI) (Sazonovs et al., 2020; Wilson et al., 2020). Due to the differences in genetic backgrounds of the populations, the association between genetic variants and ATI production in Chinese populations needs to be confirmed.

Variations in genes coding for TNF, TNF receptor superfamily 1A (TNFRSF1A), TNF receptor superfamily 1B (TNFRSF1B) and a receptor for the Fc portion of IgG (FcγRIIIa) were recently associated with the response to anti-TNF-α drugs in CD (Louis et al., 2006; Linares-Pineda et al., 2018; Matsuoka et al., 2018; Romero-Cara et al., 2018; Curci et al., 2021). The association between genetic polymorphisms in the *ATG16L1* gene contributing to CD risk and response to anti-TNF-α treatment suggests that genetic variants involved in the susceptibility to chronic inflammatory diseases plays an important role in the drug response (Koder et al., 2015). Genetic variations in these aforementioned genes may affect response to IFX through ADA formation*.* None of all these single nucleotide polymorphisms (SNPs) within the relevant genes involved in the susceptibility to chronic inflammatory diseases, cytokines and apoptosis pathways have yet been investigated at the same time with the aim of identifying patients who develop ATI. Based on the frequency of SNPs in Chinese populations, 13 SNPs in 13 genes were selected to investigate the association between genetic variants and development of ATI*.*

The present study assessed the association between genetic variants and ATI production during the IFX maintenance therapy in Chinese patients with CD, offering evidence of the risk of ATI production as well as new opportunities for stratifying patients based on their genetic makeup prior to initiation of IFX therapy.

## Materials and methods

### Patients and samples

Patients with CD were enrolled between October 2020 and April 2022 at the Department of Gastroenterology, the First Affiliated Hospital of Soochow University (Suzhou, China). Participants (>18 years old) had a histopathological diagnosis of CD and received scheduled therapy. The treatment regimen included the administration of 5 mg/kg IFX at weeks 0, 2, and 6 and then every 8 weeks during maintenance therapy. A stable dose of 5-aminosalicylic acid (5-ASA) was allowed for at least 4 weeks. The study excluded patients who had been administered IFX for more than 2 years and had comedications including immunomodulators (azathioprine, 6-mercaptopurine) and steroids. Electronic medical records were used to collect information about weight, age, sex, smoking history, disease phenotype and faecal calprotectin (fCal) values. Within the 24 h before IFX infusion, a total of 2 mL of peripheral whole blood was collected for genetic analysis as well as TLI and ATI measurements. Meanwhile, the Crohn’s disease activity index (CDAI) was recorded. Clinical remission (CR) was defined as a CDAI less than 150 points. The normalization of fCal was defined as less than 250 μg/g. Patients provided written and informed consent. The research was approved by the Research Ethics Committee of the First Affiliated Hospital of Soochow University.

### Measurement of infliximab levels

Separation of plasma from whole blood was performed to detect TLI. TLI were analyzed with an in-house developed and validated ELISA method. High binding 96-well plates were coated overnight with TNF-α (300-01A, PeproTech, United Kingdom) at 4°C. Plasma samples were diluted in PBS and incubated for 2 h at 37°C in a shaker. Human anti-infliximab, clone AbD19376_hIgG1 (HCA216P, Bio-Rad, United Kingdom), was used as the detecting agent. The lower and upper limits of quantification were 0.50 μg/mL and 40.00 μg/mL, respectively.

### Detection of antibodies to infliximab

ATI was detected by a bridging ELISA method. The analytical method was validated according to current industry practice (Myler et al., 2021). Plasma samples and controls were diluted in glycine (100 mM, pH 2.5) at a minimum dilution of 1:20 for 30 min at RT in a shaker. Then, 50 μL of the diluted samples or controls and 95 μL master mixture containing 0.1 μg/mL each of biotin-IFX, HRP-IFX and 5 μL of Tris (1.5 M, pH 9.0) were added to a 96-well polypropylene plate and then incubated for 2 h at RT in a shaker. Immediately after, 100 μL of a mixture was moved to a streptavidin matrix coated 96-well plate and incubated at RT for 1 h in a shaker. The plate was washed three times, followed by the addition of 3,3’,5,5’-tetramethylbenzidine substrate and 2 M H_2_SO_4_. A negative control (NC) of pooled plasma from IFX-naïve CD patients as well as low and high positive controls containing 100 and 20, 000 ng/mL rabbit anti-IFX polyclonal antibodies prepared in NC, was included in each plate. The screening cut point based on a panel of plasma samples from 51 IFX-naïve CD patients, was determined to be 1.16 (S/N). Based on the analysis of 51 IFX-naïve plasma samples from CD patients without or with IFX (20 μg/mL), the confirmatory cut point was determined to be a decrease in signal of more than 17.21%. The titer cut point was determined to be 1.34. The minimum significant ratio was determined to be 3. ATI titers of 1:20 and ≥1:60 were considered a low titer and a high titer, respectively. Drug-tolerance at low positive control was ≤12.50 μg/mL IFX. The relative sensitivity of this assay was determined to be 50 ng/mL (for validation parameters, see Supplementary Table S1).

### DNA extraction and genotypic analysis

DNA was extracted from 250 μL of whole blood using a DNA Extraction Kit (Takara, Japan). The concentration and purity of DNA were calculated with the OD-1000 (OneDrop^TM^, China). Thirteen primer pools of SNPs, namely, *HLA-DQA1* (rs2097432), *FCGR3A* (rs396991), *FCGR2A* (rs1801274)*, PTPRC* (rs10919563)*, KLRC1* (rs7301582)*, HLA-E* (rs1264457), *IL-17RA* (rs4819554), *ATG16L1* (rs10210302), *TRAF1* (rs3761847), *TNF* (rs1800629), *TNFRSF1A* (rs767455), *TNFRSF1B* (rs1061622) and *CCNY* (rs12777960) (Supplementary Table S2), were designed. Library preparation was performed using a two-step polymerase chain reaction (PCR). These reaction mixtures contained 1 μL of DNA, 0.4 mM dNTPs, 3 μM of each primer, Taq Buffer (with MgCL_2_) and 0.04 U Taq DNA polymerase. Twenty-five μL of reaction mixture was used for PCR. The first PCR conditions were 95°C for 5 min, followed by 10 cycles of 94°C for 30 s, 63°C for 30 s, and 72°C for 30 s. The second PCR conditions were 30 cycles of 95°C for 30 s, 58°C for 30 s, and 72°C for 30 s, with a final extension at 72°C for 10 min. All PCR reagents were provided by ThermoFisher (Waltham, MA) and Sangon Biotech (Shanghai, China). Paired-end sequencing of the library was performed on HiSeq XTen sequencers (Illumina, San Diego, CA).

### Statistical analysis

Statistical analysis was performed using GraphPad Prism® version 9.0 and IBM SPSS Statistics for Mac, version 26.0. The TLI below the lower limits of quantification were imputed by 0.25 μg/mL. The mean and standard deviation or the median and interquartile range (IQR) were used to describe continuous clinical and demographic variables. Categorical variables were expressed as percentages. The Mann‒Whitney U test (two groups) and the Kruskal–Wallis H test (more than two groups) were used to compare continuous non-normality variables. The distribution of 13 SNPs within 13 genes was tested for Hardy-Weinberg equilibrium using the chi-squared goodness-of-fit test. For all SNPs, two genetic models were used: the additive model, which supposes that the effect of the risk allele increases with the addition of each allele copy; and the dominant model, which analyzes the heterosis. A chi-squared test and logistic regression were performed to evaluate the association and multiplicative interactions between SNPs and ATI formation, respectively. All reported *p* values were 2-sided, and a significant *p* < .05 was considered a statistically significant difference.

## Results

### Study population

A total of 104 patients with CD were included. The patients’ demographic and clinical characteristics are shown in Table 1. A total of 73.08% of patients were male. The median duration of IFX treatment was 10 months (IQR: 5–16.5). The median CDAI point was 87.6 (IQR: 68.2–123.5). The median fCal value was 64.5 (IQR: 30–344.5). The median TLI in CD patients was 2.89 μg/mL (IQR: 1.58–5.07). The TLI in 10 patients was less than 0.50 μg/mL (10/104, 9.62%).

**TABLE 1:** Patients’ demographic and clinical characteristics.

Patient’s demographic and clinical characteristics	N = 104
Male, (%)	76 (73.08)
Age at diagnosis, median (IQR), y	29 (23–33)
Age at IFX initiation (years), median (IQR)	30 (24–37)
Duration of IFX therapy (months), median (IQR)	10 (5–16.5)
Location^a^: L1/L2/L3/L4a	55/13/35/1
Behaviour^a^: B1/B2/B3	57/39/8
Non-smoker, (%)	84 (80.77)
Previous surgery, (%)	19 (18.27)
Prior IFX use, (%)	6 (5.77)
CDAI points, median (IQR)	87.6 (68.2–123.5)
Faecal calprotectin (μg/g), median (IQR)	64.5 (30–344.5)
Albumin (g/L), median (IQR)	43.6 (41.1–46.1)
ESR (mm/H), median (IQR)	2 (1–5)
Trough levels of infliximab (μg/mL), median (IQR)	2.89 (1.58–5.07)

^a^ According to the Montreal classification.

y, years; IFX, infliximab; CDAI, Crohn's disease activity index; ESR, erythrocyte sedimentation rate; IQR, interquartile range.

### Higher TLI increases the probability of therapeutic success

**FIGURE 1:**
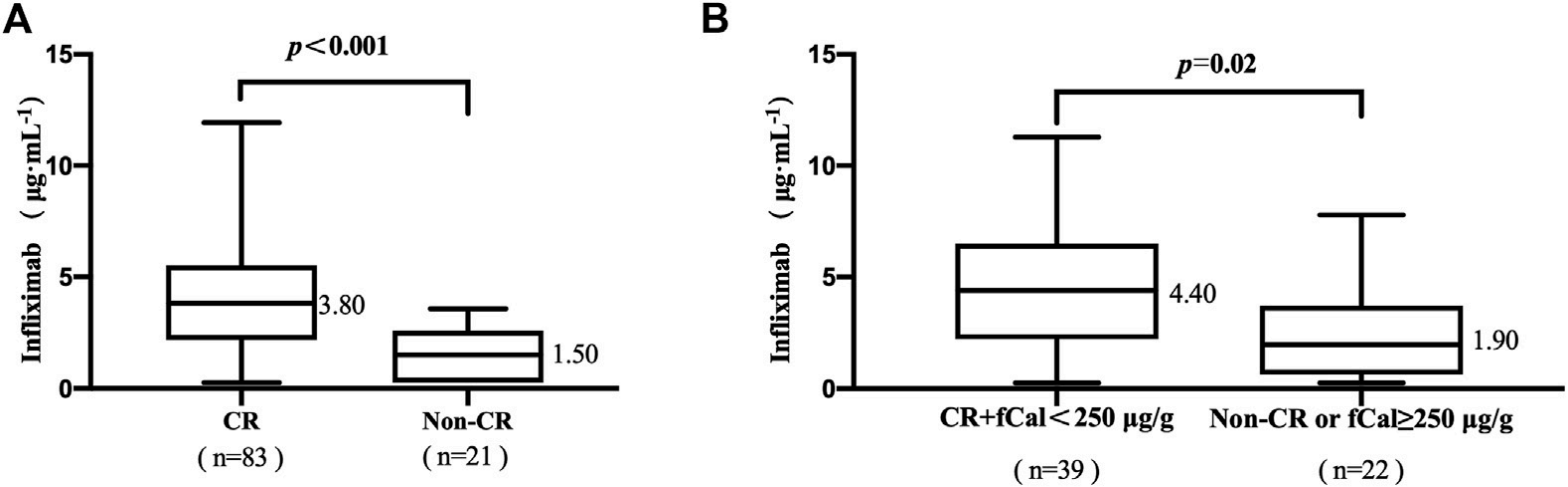
**(A)** Association between trough levels of infliximab (TLI) and clinical remission (CR). **(B)** The association of TLI with CR and faecal calprotectin (fCal) normalization. Clinical remission (CR) was defined by a Crohn’s disease activity index (CDAI) < 150 points. Normalization of faecal calprotectin (fCal) was defined if below 250 μg/g stools. Data for fCal were not available for all patients included in this study.

Eighty-three of the 104 patients (79.81%) achieved clinical remission (CR). The median TLI in patients with CR was higher than that in patients without CR (3.80 vs. 1.50 μg/mL, *p* < .001, Figure 1A). The median TLI in patients who achieved CR and fCal normalization was higher than that in patients who did not (4.40 vs. 1.90 μg/mL, *p =* .02, Figure 1B).

### Relationships between TLI and titers of ATI

**FIGURE 2:**
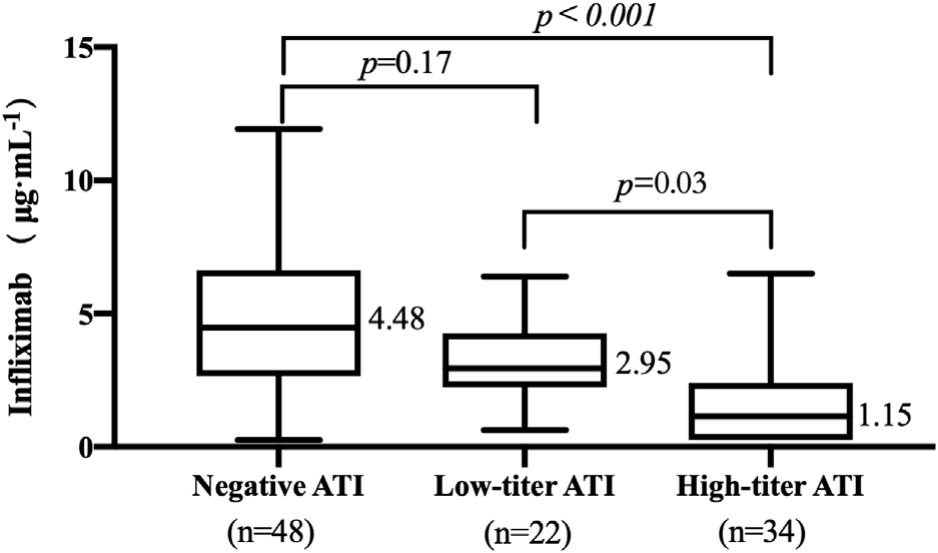
Relationship between trough levels of infliximab (TLI) and antibodies to infliximab (ATI) titer. ATI titers of 1:20 and ≥1:60 were considered a low titer and a high titer, respectively.

The TLI ranged from undetectable to 11.93 μg/mL. The occurrence of ATI was 53.85% (56/104). The titers of ATI showed a wide range from low to high titers. The relationship between TLI and titers of ATI was evaluated. The median TLI in patients with high-titer ATI (1.15 μg/mL, IQR: 0.25–2.39, Figure 2) was significantly lower than that in patients with low-titer ATI (2.95 μg/mL, IQR: 2.25–4.28, *p* = .03, Figure 2). A significant difference in TLI was also observed between the high-titer group and the negative-ATI group (4.48 μg/mL, IQR: 2.65–6.64, *p <* .001, Figure 2). No significant differences in TLI were observed between the negative-ATI group and the low-titer group (*p* = .17, Figure 2).

### Association between genetic variants and ATI production

**FIGURE 3:**
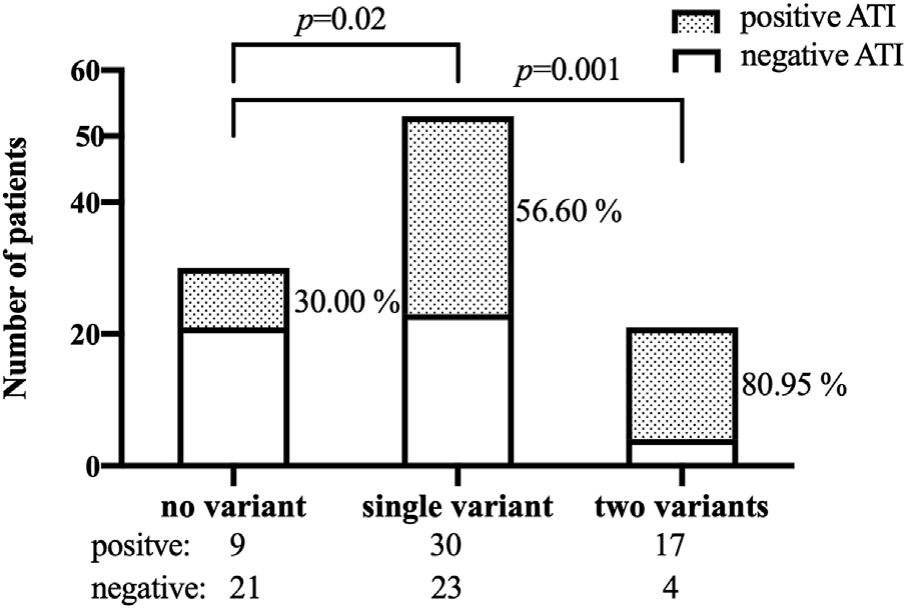
Patients who developed ATI according to the number of the variants in both SNPs (rs2097432 and rs393991). ATI: antibodies to infliximab.

The frequencies of genotypes for 13 SNPs within 13 genes in 104 patients were in line with Hardy-Weinberg equilibrium (HWE, *p* > .05, Supplementary Table S3 in the Supplement). The association between 13 SNPs and ATI formation under the dominant model and additive model are shown in Supplementary Table S4. Variant carriers of the SNP (rs2097432 in HLA-DQA1*05) under the dominant model had an higher rate of ATI formation (GG and AG: 27/38 = 71.05%, AA: 29/66 = 43.94%, *p* = .01, Table 2). There was also a significant association between rs396991 in *FCGR3A* and ATI production under the dominant model. Carriers of the variant SNP (rs396991 in *FCGR3A*) had a higher probability of producing ATI (CC and AC:37/57 = 64.91%, AA: 19/47 = 40.43%, *p* = .01, Table 2). No association was found between other SNPs and ATI production (Table 2; Supplementary Table S5). In logistic regression, the HLA-DQA1*05 rs2097432 GG and GA genotypes increased the risk of ATI formation compared to the AA genotype (GG and AG vs. AA, OR 2.94, 95% CI 1.19–7.30, *p* = .02, Table 3). Patients carrying the CC and AC genotypes of rs396991 in *FCGR3A* were associated with a higher frequency of ATI formation (CC and AC vs. AA, OR 2.94, 95% CI 1.24–6.96, *p* = .01, Table 3). According to the number of the variants in rs2097432 and rs393991, a greater proportion of patients with two variants showed ATI production (two variants vs. no variant, 17/21 = 80.95% vs. 9/30 = 30.00%, OR 9.92, 95% CI 2.59–37.87, *p* = .001; single variant vs. no variant, 30/53 = 56.60% vs. 9/30 = 30.00%, OR 3.04, 95% CI 1.18–7.88, *p* = .02, Figure 3 and Table 3).

**TABLE 2:** Patients who developed antibodies to infliximab according to single nucleotide polymorphisms (SNPs) under IKthe dominant model.

Gene	Rs number	Genotype	Case	Positive ATI (%)	*p*
Immune processes					
*HLA-DQA1*	rs2097432	AA	66	29 (43.94)	.01
		GG + GA	38	27 (71.05)	
*FCGR3A*	rs396991	AA	47	19 (40.43)	.01
		CC + AC	57	37 (64.91)	
*FCGR2A*	rs1801274	AA	43	24 (55.81)	.74
		AG + GG	61	32 (52.46)	
Susceptibility to chronic inflammatory diseases					
*PTPRC*	rs10919563	GG	59	30 (50.85)	.48
		GA + AA	45	26 (57.78)	
*KLRC1*	rs7301582	CC	79	41 (51.89)	.48
		CT + TT	25	15 (60.00)	
*HLA-E*	rs1264457	GG	40	20 (50.00)	.53
		GA + AA	64	36 (56.25)	
*IL17RA*	rs4819554	AA	32	15(46.88)	.34
		GG + GA	72	41(56.94)	
*ATG16L1*	rs10210302	CC	35	20(57.14)	.63
		CT + TT	69	36 (52.17)	
*TRAF1*	rs3761847	AA	35	18 (51.43)	.73
		GA + GG	69	38 (55.07)	
Cytokines					
*TNF*	rs1800629	GG	88	47 (53.41)	.83
		GA + AA	16	9 (56.25)	
*TNFRSF1A*	rs767455	TT	85	46 (54.12)	.95
		CC + TC	19	10 (52.63)	
*TNFRSF1B*	rs1061622	TT	75	42 (56.00)	.48
		GG + TG	29	14 (48.28)	
Apoptosis					
*CCNY*	rs12777960	CC	40	21 (52.50)	.83
		CA + AA	64	35 (54.69)	

ATI: antibodies to infliximab.

**TABLE 3:** Logistic regression: the development of antibodies to infliximab (ATI).

SNPs	Genotype	Odds ratio	95% Confidence interval	*p*
rs2097432	AA	1		
	GG + GA	2.94	1.19–7.30	.02
rs396991	AA	1		
	CC + AC	2.94	1.24–6.96	.01
The combinations of rs2097432 and rs396991	AA + AA	1		
	AA + AC/CC or GA/GG + AA	3.04	1.18–7.88	.02
	GA + AC/CC or GG + AC/CC	9.92	2.59–37.87	.001

## Discussion

The production of ATI constitutes an important cause of subtherapeutic levels of IFX. Therefore, patients at risk of ATI formation should be identified. In this cross-sectional study, the association between ATI production and the SNPs in the genes involved in several pathways was assessed in Chinese patients with CD, demonstrating that patients with two of the variants in rs2097432 and rs393991 had an almost 10-fold higher risk of producing ATI than those with no variant.

Using a drug-tolerant ATI assay, the present study showed that HLA-DQA1*05 and *FCGR3A* genetic variation are associated with ATI formation in Chinese patients with CD, in line with the previous studies in Europe and Canada (Romero-Cara et al., 2018; Sazonovs et al., 2020; Wilson et al., 2020; Curci et al., 2021). The HLA-DQA1*05 genotype is also associated with the development of antibodies to adalimumab, IFX’s sister TNF antagonist (Powell Doherty et al., 2020). HLA may affect antigen binding and immune cell activation downstream (Choo, 2007). The mechanisms of HLA-DQA1*05 variants in ATI formation should be further investigated. Another gene involved in the immune process, the Fc-gamma receptors (FcγR) type IIIA (*FCGR3A*), is able to remove antigen-antibody complexes from the circulation. *FCGR3A* variant carriers have a higher binding affinity of FcγRs to IgG as well as greater antibody-dependent cellular cytotoxicity activity (Moroi et al., 2013).

Our study did not find the association between ATI production and polymorphisms in the genes involved in the susceptibility to chronic inflammatory diseases, cytokines and apoptosis pathways. First, the limited sample size may not provide sufficient statistical power to determine the effect of 11 SNPs on the ATI formation. Perhaps in a larger sample study, we can find obvious associations between those SNPs and ATI production. Second, some other mechanisms of these 11 SNPs in the IFX response should be investigated. Third, the distribution of the *TNFRSF1A* and *IL17RA* genotypes in Chinese populations differed from that in European populations (Supplementary Table S3).

The rates of ATI production across different assays have varied widely from 0% to 79% (Gorovits et al., 2018). Most ATI detection assays are drug sensitive (Barrau et al., 2022). Using a drug-tolerant ATI assay, the positive rate of ATI increased from 21% (drug-sensitive ATI assay) to 63% (Van Stappen et al., 2018). Therefore, among these studies, drug-sensitive ATI assays may lead to an underestimated the rate of ATI formation (Romero-Cara et al., 2018; Wilson et al., 2020; Curci et al., 2021). The dissociation of drug/anti-drug antibodies complexes with acid and subsequent detection of ADA have been successfully applied to improve drug tolerance (Patton et al., 2005; Sickert et al., 2008; Mikulskis et al., 2011). Using acid dissociation in most assays, though others using anti-lambda chain antibody, or more rarely heat-dissociation allows the assay to be drug-tolerant. In the present study the dissociation of IFX/ATI complexes using glycine and subsequent detection of ATI by bridging assays improved drug tolerance. The drug tolerance in our assay was determined to be 12.5 μg/mL when the low positive control was at 100 ng/mL, which is higher than the highest TLI (11.93 μg/mL). This method has the ability to detect multivalent monospecific antibodies such as IgG or IgM antibodies but not bispecific antibodies such as IgG4.

There are limitations that need to be considered. The results were based on a cross-sectional study, and a prospective cohort study with a larger sample size is therefore needed. Additionally, as well as the presence or absence of ATI, the duration of the response (transient, persistent) should also be considered. Transient vs. sustained ATI were not differentiated in this work. Transient ATI may become negative with no clinically meaningful impact. Although these limitations exist, the discovery of rs2097432 in HLA-DQA1*05 and rs396991 in *FCGR3A* provides further evidence that genotyping may optimize IFX treatment in Chinese patients with CD.

In summary, rs2097432 in HLA-DQA1*05 and rs396991 in *FCGR3A* are significantly associated with the development of ATI. Patients with two variants (rs2097432 and rs393991) had an almost 10-fold risk of producing ATI than those with no variants. To achieve personalized therapy in CD patients treated with IFX, genetic testing in genes involved in ATI formation prior to initiation of IFX therapy, such as rs2097432 in HLA-DQA1*05 and rs396991 in *FCGR3A*, could be suggested.

## Data Availability

The datasets presented in this study can be found in online repositories. The names of the repository/repositories and accession number(s) can be found below: http://www.ensembl.org/index.html, rs396991 and rs2097432, https://www.ncbi.nlm.nih.gov/snp/, rs396991 and rs2097432.
