# Supplementary material for: Association between genetic variants and development of antibodies to infliximab: A cross-sectional study in Chinese patients with Crohn’s disease
Source: Front Pharmacol. 2023 Jan 16;14:1096816. doi: 10.3389/fphar.2023.1096816 (PMC9885127; doi:10.3389/fphar.2023.1096816)
Supplement: Supplementary file 1 [file DataSheet1.docx]

Supplement Table S1. Summary of key validation parameters for the detection of antibodies to infliximab (ATI).

| Validation Parameters | Results |
| --- | --- |
| Screening cut point (SCP) | 1.16 (S/N) |
| Confirmatory cut point (CCP) | 17.21% |
| Titer cut point (TCP) | 1.34 |
| Minimum significant ratio(MSR) | 2.6 |
| The concentration of high positive control | 20,000 ng/mL |
| The concentration of low positive control | 100 ng/mL |
| Intra-assay and inter-assay screening assay performance | Precision, CV (%) |
| Low positive control (LPC) | 8.22 (intra-assay) / 13.74 (inter-assay) |
| Middle positive control | 8.21(intra-assay) / 18.53 (inter-assay) |
| High positive control (HPC) | 10.09 (intra-assay) / 19.36 (inter-assay) |
| Intra-assay and Inter-assay Confirmatory assay Performance | Precision, CV (%) |
| Low positive control | 4.83 (intra-assay) / 12.43 (inter-assay) |
| Middle positive control | 3.71 (intra-assay) / 4.39 (inter-assay) |
| High positive control | 0.02 (intra-assay) / 0.08 (inter-assay) |
| Assay sensitivity | 50 ng/mL (SCP and CCP) |
| Selectivity | Blank (9/10 met criteria, SCP; 9/10 met criteria, CCP)  LPC (10/10 met criteria, SCP; 10/10 met criteria, CCP) |
| Hemolysis | Blank (5/5 met criteria, SCP; 4/5 met criteria, CCP)  LPC (5/5 met criteria, SCP; 5/5 met criteria, CCP) |
| Lipemia | Blank (5/5 met criteria, SCP; 5/5 met criteria, CCP)  LPC (5/5 met criteria, SCP; 5/5 met criteria, CCP) |
| Minimum required dilution (MRD) | 1 in 20 |
| Prozone | not present up to 100,0000 ng/mL |
| Drug tolerance | 12.5 μg/mL (100 ng/mL, SCP and CCP)  25 μg/mL (250 ng/mL, SCP and CCP) |
| Confirmatory drug concentration | 20,000 ng/mL |
| Stability | |
| Room temperature | up to 24 hr |
| Freeze-thaw | up to five freeze-thaw cycles |
| 4℃ | up to 1 week |

Supplement Table S2. PCR primers.

| Gene | Rs number | Foward (5’-3’) | Reverse (5’-3’) |
| --- | --- | --- | --- |
| Immune processes | | | |
| *HLA-DQA1* | rs2097432 | TATGTCATCATCCTTCCCCTTCAAC | ATCACAGCCAGGAGTCCTTAACTAT |
| *FCGR3A* | rs396991 | TCACATATTTACAGAATGGCAAAGG | TCCCAACTCAACTTCCCAGTGTGAT |
| *FCGR2A* | rs1801274 | TGGTTTGCTTGTGGGATGGAGAAGG | TCTGCCAGACATCATGTCAAGTTCT |
| Susceptibility to chronic inflammatory diseases | | | |
| *PTPRC* | rs10919563 | TGTACTTCAGATCTCTTCTGGGCTT | GTCATGGGTATAAGGGTATTAATCA |
| *KLRC1* | rs7301582 | GTAAGATGTAAATGTTTTAAACACT | TGTATTGTGGATTGAAAGCATTTCT |
| *HLA-E* | rs1264457 | TCAGAGGCATCATTTGACTTTTGCT | TTCTTTTTCAGTTTAGGCCAAAATG |
| *IL-17RA* | rs4819554 | CTCTTTCTCAAGACAAGGACAGCTC | GTGCTAAGAAGGAGACTCATGAAAT |
| *ATG16L* | rs10210302 | ATGAAAATGGAGCCAGACAAAGTAG | CTTTTTCATTTTCTTCAGCGTGTGA |
| *TRAF1* | rs3761847 | ACGTTTTGATGTCCGTGGGAATGAG | CATCTTACCGCCACAATGTACATCA |
| Cytokines | | | |
| *TNF* | rs1800629 | GGGACACACAAGCATCAAGGATACC | CCTCCCAGTTCTAGTTCTATCTTTT |
| *TNFRSF1A* | rs767455 | GGGGAGTGAGAGGCCATAGCTGTCT | GCCCCTACTCCAAAAGGCGGATGAA |
| *TNFRSF1B* | rs1061622 | AATGAGACTTCTTGCGACTTACCAA | GCCTCCTCCTCCTCCAGCTGTAACG |
| Apoptosis | | | |
| *CCNY* | rs12777960 | TGCAGTGTAGTAATGTGTTAAAAGG | CCCTCTAGAGGAGGTGCTTTTCTAT |

Supplement Table S3. The results of the Hardy Weinberg equilibrium analysis in 13 SNPs.

| Gene | Rs number | Genotype | N | Minor  Allele | MAF(%) | | Most severe consequence ^a^ | | P   value |
| --- | --- | --- | --- | --- | --- | --- | --- | --- | --- |
|  |  |  |  |  | Chinese | European^b^ |  |  |  |
| Immune processes | | | | | |  |  | |  |
| *HLA-DQA1* | rs2097432 | GG,GA,AA | 3,35,66 | G | 19.71 | 27.11 | intergenic variant | | 0.81 |
| *FCGR3A* | rs396991 | CC,AC,AA | 14,43,47 | C | 34.13 | 31.69 | missense | | 0.71 |
| *FCGR2A* | rs1801274 | GG,AG,AA | 21,40.43 | G | 39.42 | 49.13 | missense | | 0.14 |
| Susceptibility to chronic inflammatory diseases | | | | | | | |  | |
| *PTPRC* | rs10919563 | GG,GA,AA | 59,40,5 | A | 24.04 | 11.98 | intron | | 0.86 |
| *KLRC1* | rs7301582 | CC,CT,TT | 79,23,2 | T | 12.98 | 21.56 | intron | | 0.98 |
| *HLA-E* | rs1264457 | GG,GA,AA | 40,45,19 | A | 39.90 | 44.53 | missense | | 0.61 |
| *IL17RA* | rs4819554 | GG,GA,AA | 18,54,32 | G | 43.27 | 19.17 | regulatory region | | 0.84 |
| *ATG16L1* | rs10210302 | CC,CT,TT | 35,40,29 | T | 47.12 | 48.39 | intron | | 0.07 |
| *TRAF1* | rs3761847 | GG,GA,AA | 15,54,35 | G | 40.38 | 42.22 | intron | | 0.73 |
| Cytokines | | | | | | | |  | |
| *TNF* | rs1800629 | GG,GA,AA | 88,15,1 | A | 8.17 | 15.93 | intergenic variant | | 0.92 |
| *TNFRSF1A* | rs767455 | CC,TC,TT | 1,18,85 | C | 9.62 | 43.38 | synonymous | | 1.00 |
| *TNFRSF1B* | rs1061622 | GG,TG,TT | 3,26,75 | G | 15.38 | 23.42 | missense | | 0.92 |
| Apoptosis |  |  |  |  |  |  |  | |  |
| *CCNY* | rs12777960 | CC,CA,AA | 40,55,9 | A | 35.10 | 32.82 | intron | | 0.26 |

^a^From Ensembl Genome Browser databases (http://www.ensembl.org/index.html).

^b^This is referred from NCBI (https://www.ncbi.nlm.nih.gov/snp/).

MAF, minor allele frequency.

Supplement Table S4. Association between 13 SNPs and ATI formation under dominant model and additive model.

| Gene | Rs number | Dominant model | | Additive model | |
| --- | --- | --- | --- | --- | --- |
|  |  | OR(95%CI) | *P* | OR(95%CI) | *P* |
| Immune processes | | | | | |
| *HLA-DQA1* | rs2097432 | 2.94(1.19-7.30) | 0.02 | 0.00 (0.00-)^*^ | 1.00 |
| *FCGR3A* | rs396991 | 2.94(1.24-6.96) | 0.01 | 1.10(0.59-2.05) | 0.75 |
| *FCGR2A* | rs1801274 | 0.87(0.39-1.91) | 0.74 | 0.72(0.25-2.05) | 0.54 |
| Susceptibility to chronic inflammatory diseases | | | | | |
| *PTPRC* | rs10919563 | 1.32(0.61-2.89) | 0.48 | 0.64(0.10-4.14) | 0.64 |
| *KLRC1* | rs7301582 | 1.39(0.56-3.47) | 0.48 | 0.93(0.06-15.34) | 0.96 |
| *HLA-E* | rs1264457 | 1.28(0.58-2.84) | 0.53 | 0.85(0.49-1.48) | 0.57 |
| *IL17RA* | rs4819554 | 1.49(0.65-3.46) | 0.34 | 0.94(0.53-1.67） | 0.83 |
| *ATG16L1* | rs10210302 | 0.82(0.36-1.86) | 0.63 | 0.89(0.55-1.40） | 0.66 |
| *TRAF1* | rs3761847 | 1.16(0.51-2.62) | 0.73 | 0.84(0.46-1.55） | 0.58 |
| Cytokines | | | | | |
| *TNF* | rs1800629 | 1.12(0.38-3.28) | 0.83 | 37539.89(0.00-)^*^ | 1.0 |
| *TNFRSF1A* | rs767455 | 0.94(0.35-2.55) | 0.95 | 43650.01(0.00-)^*^ | 1.0 |
| *TNFRSF1B* | rs1061622 | 0.73(0.31-1.73) | 0.48 | 0.79(0.24-2.71） | 0.72 |
| Apoptosis |  |  |  |  |  |
| *CCNY* | rs12777960 | 1.09(0.49-2.41) | 0.83 | 1.34(0.63-2.87） | 0.44 |

OR: Odds ratio; CI: confidence interval; *: the limited sample size did not provide sufficient statistical power to determine the effect; ATI: antibodies to infliximab.

Supplement Table S5. Logistic regression: the development of antibodies to infliximab (ATI).

| Gene | SNPs | Genotype | Odds ratio | 95%  Confidence interval | *P* |
| --- | --- | --- | --- | --- | --- |
| Immune processes | | | | | |
| *FCGR2A* | rs1801274 | AA | 1 |  |  |
|  |  | AG+GG | 0.87 | 0.39-1.91 | 0.74 |
| Susceptibility to chronic inflammatory diseases | | | | | |
| *PTPRC* | rs10919563 | GG | 1 |  |  |
|  |  | GA+AA | 1.32 | 0.61-2.89 | 0.48 |
| *KLRC1* | rs7301582 | CC | 1 |  |  |
|  |  | CT+TT | 1.39 | 0.56-3.47 | 0.48 |
| *HLA-E* | rs1264457 | GG | 1 |  |  |
|  |  | GA+AA | 1.29 | 0.58-2.84 | 0.53 |
| *IL17RA* | rs4819554 | AA | 1 |  |  |
|  |  | GG+GA | 1.49 | 0.65-3.46 | 0.34 |
| *ATG16L1* | rs10210302 | CC | 1 |  |  |
|  |  | CT+TT | 0.82 | 0.36-1.86 | 0.63 |
| *TRAF1* | rs3761847 | AA | 1 |  |  |
|  |  | GA+GG | 1.16 | 0.51-2.62 | 0.73 |
| Cytokines | | | | | |
| *TNF* | rs1800629 | GG | 1 |  |  |
|  |  | GA+AA | 1.12 | 0.38-3.28 | 0.83 |
| *TNFRSF1A* | rs767455 | TT | 1 |  |  |
|  |  | CC+TC | 0.94 | 0.35-2.55 | 0.95 |
| *TNFRSF1B* | rs1061622 | TT | 1 |  |  |
|  |  | GG+TG | 0.73 | 0.31-1.73 | 0.48 |
| Apoptosis | | | | | |
| *CCNY* | rs12777960 | CC | 1 |  |  |
|  |  | CA+AA | 1.09 | 0.49-2.41 | 0.83 |
